# *In vivo* label-free measurement of lymph flow velocity and volumetric flow rates using Doppler optical coherence tomography

**DOI:** 10.1038/srep29035

**Published:** 2016-07-05

**Authors:** Cedric Blatter, Eelco F. J. Meijer, Ahhyun S. Nam, Dennis Jones, Brett E. Bouma, Timothy P. Padera, Benjamin J. Vakoc

**Affiliations:** 1 Wellman Center for Photomedicine, Massachusetts General Hospital, Boston, Massachusetts 02114, USA; 2 Harvard Medical School, Boston, Massachusetts 02115, USA; 3 Edwin L. Steele Laboratories for Tumor Biology, Department of Radiation Oncology, Massachusetts General Hospital Cancer Center, Boston, Massachusetts 02114, USA; 4 Department of Mechanical Engineering, Massachusetts Institute of Technology, 77 Massachusetts Avenue, Cambridge, Massachusetts 02139, USA

## Abstract

Direct *in vivo* imaging of lymph flow is key to understanding lymphatic system function in normal and disease states. Optical microscopy techniques provide the resolution required for these measurements, but existing optical techniques for measuring lymph flow require complex protocols and provide limited temporal resolution. Here, we describe a Doppler optical coherence tomography platform that allows direct, label-free quantification of lymph velocity and volumetric flow rates. We overcome the challenge of very low scattering by employing a Doppler algorithm that operates on low signal-to-noise measurements. We show that this technique can measure lymph velocity at sufficiently high temporal resolution to resolve the dynamic pulsatile flow in collecting lymphatic vessels.

The lymphatic system drains fluid and metabolic waste from tissues and provides a route for antigen and antigen presenting cells to move from tissue to lymph nodes^1^. When the lymphatic system becomes dysfunctional, whether as a result of trauma, surgery, infection, or other causes, the body’s ability to maintain fluid balance and tissue homeostasis is compromised and immune function is impaired. This manifests clinically as lymphedema^2^. Current treatments for lymphedema are limited to palliative measures, such as massage or compression garments, rather than direct repair of the damaged lymphatic system, which would likely be more effective and longer lasting. To this end, preclinical investigations are focused on identifying the biological and molecular regulators of lymph transport in normal and disease settings^3^. In these studies, accurate *in vivo* measurements of lymph flow velocity and lymph volumetric flow will be critical.

Unfortunately, *in vivo* assessment of lymph transport has proven extremely challenging. Because the lymphatic network is filled from the periphery, it is difficult to globally label lymph. Instead, regional labeling is performed by injecting a radioactive, fluorescent or chromatic dye into the tissue and imaging the draining lymphatic vessels^4^,^5^,^6^,^7^,^8^. While these methods are effective for lymphangiography, i.e., for imaging the morphology of the lymphatic network, their utility for measuring flow and function is limited by several factors. First, the injection of the label alters the interstitial fluid pressure in the tissue, which in turn perturbs the physiological state of the lymphatic system. Thus the method intrinsically changes the parameters to be measured. Second, label-based techniques offer imperfect measurements of flow and are often complex to implement. For example, it is possible to estimate the lymphatic transport by tracking the movement of the fluorescent label front after injection^9^. However, this approach cannot reveal pulsatile flow dynamics and is valid only during the brief initial filling of the targeted vessel. Continuous measurements can be performed by tracking photobleached spots induced by a high-intensity beam. However, these methods are limited by label diffusion and generally yield low-fidelity signals with limited temporal resolution^10^. To avoid the perturbation induced by label-based approaches, methods based on cell tracking within lymphatic vessels have been demonstrated^11^. However, the flow measurements provided by these approaches are intermittent (occurring only when a cell is located within the field of view) and are confounded by the interaction of the cells with the lymphatic endothelium^12^. Finally, lymph “packet” velocity can be measured with near infrared fluorescence techniques. These measurements have been shown to be useful, but are able to only characterize one intermittent aspect of lymph flow dynamics^5^,^7^,^13^,^14^,^15^. Because of these technical limitations, the nature and regulation of lymph flow *in vivo* is poorly understood.

Here, we demonstrate direct label-free measurement of lymph flow *in vivo* by Doppler optical coherence tomography (OCT). OCT systems provide spatially localized measurements of optical scattering within tissue^16^. Doppler OCT (DOCT) systems additionally measure the motion of the scatterers and can be used to quantify fluid flow velocity^17^,^18^,^19^,^20^. For example, quantitative blood flow imaging by DOCT has been demonstrated in preclinical^21^ and clinical^22^ applications. However, there are no reports showing DOCT-based measurement of lymph flow. The challenge is not the slower speed of lymph flow relative to blood flow, but rather the profoundly different optical properties of the fluids. Blood is highly scattering and thus produces a significant OCT signal. Lymph is nearly transparent^23^,^24^,^25^,^26^; its OCT signal is approximately 20 dB lower than that of blood (Supplementary Fig. 1). Even in optimal imaging conditions, this causes lymph signals to be near the noise floor of the instrument. In this work, we demonstrate that a Doppler shift can be detected from these extremely small lymph signals. Using the Doppler OCT method, we demonstrate the first continuous *in vivo* measurement of lymph flow with temporal resolutions sufficient to quantify complex pulsatile dynamics. In addition, we demonstrate that the method can also be used to simultaneously measure lymph volumetric flow rates, e.g., measured in μL/h, and lymphatic vessel contraction. The accuracy of the measured flow velocity is confirmed by comparing DOCT and fluorescence photobleaching measurements acquired simultaneously in phantoms and *in vivo* using a multimodal microscope.

## Results

### Doppler OCT Algorithm Design

**Figure 1:**
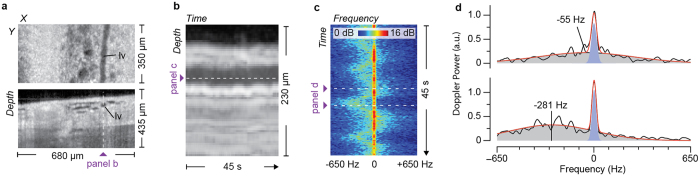
Illustration of the method to measure lymphatic flow velocity with DOCT under low SNR settings and in the presence of artifacts from neighboring static tissue signals. (**a**) Three-dimensional OCT datasets viewed in *en face* (upper panel) and cross sectional (lower panel) presentations are used to identify lymphatic vessels and select the location for flow measurement. (**b**) The depth-resolved OCT signal at a fixed transverse (x,y) location is recorded for five minutes and used to generate an M-Mode intensity image to identify the lymphatic vessel upper and lower boundaries. Depths within the lymphatic vessel are analyzed using Doppler methods. (**c**) A spectrogram is obtained for each depth, here shown for the depth indicated by the dashed line in (**b**). (**d**) Spectra showing the static and lymph signals at times of small (upper panel, −55 Hz) and moderate (lower panel, −281 Hz) Doppler shifts. Each spectral curve (black trace) within the spectrogram (panel c) is fit to a parametric model comprising two Gaussians and a white noise background (red trace). One Gaussian represents the static component and is centered at 0 Hz (blue peak). The second Gaussian represents the component due to lymph flow and is a broad distribution centered on the Doppler frequency (grey peak). The sign of the Doppler frequency shift denotes the direction of flow relative to the imaging beam.

Our algorithm operates on a set of repeated OCT measurements acquired from a fixed location within a lymphatic vessel. A three-dimensional OCT image is used to identify the vessel and select the location for flow imaging (Fig. 1a). After acquisition of the repeated measurements, the magnitude of the scattering as a function of depth and time (Fig. 1b) is used to identify the upper and lower boundaries of the lymphatic vessel (methods). The scattering signals at each depth within the lymphatic vessel are then independently analyzed to extract the lymph Doppler signal. Here, it is important that the algorithm used to extract this Doppler signal be tailored to the unique properties of *in vivo* lymph signals. We found that most lymph measurements contain a significant time-invariant signal, likely resulting from nearby highly scattering tissue structures. The magnitude of these static signals is often comparable or higher than that of lymph signals, and cause traditional Doppler methods to fail (Supplementary Fig. 2).

To separate the lymph-specific and static Doppler signals, we employed a modified joint spectral and time domain method^27^. The spectrogram of the complex scattering signal is calculated using a short-time Fourier transform (STFT) (methods, Fig. 1c). In the frequency domain, it is possible to separate the static and lymph signals. The static signal is narrowband and centered at DC (i.e., 0 Hz) while the lymph induces a spectrally broad signal that shifts in proportion to flow velocity. A white noise background is additionally present. To quantify the Doppler properties of the lymph signal separately from the static and noise signals, we fit each spectrum within the spectrogram to a parametric model comprising two Gaussians and a white noise background (methods, Fig. 1d). The center position of the Gaussian describing the spectrally broad signal estimates the Doppler shift induced by lymph flow. To calculate the flow velocity from a measured Doppler shift, it is necessary to know the angle of the velocity vector relative to the imaging beam. This is calculated using the three-dimensional scan data used to initially identify the lymphatic vessel and the flow measurement location (methods, Supplementary Fig. 3).

### Validation of DOCT Lymph Flow Velocity Measurements

**Figure 2:**
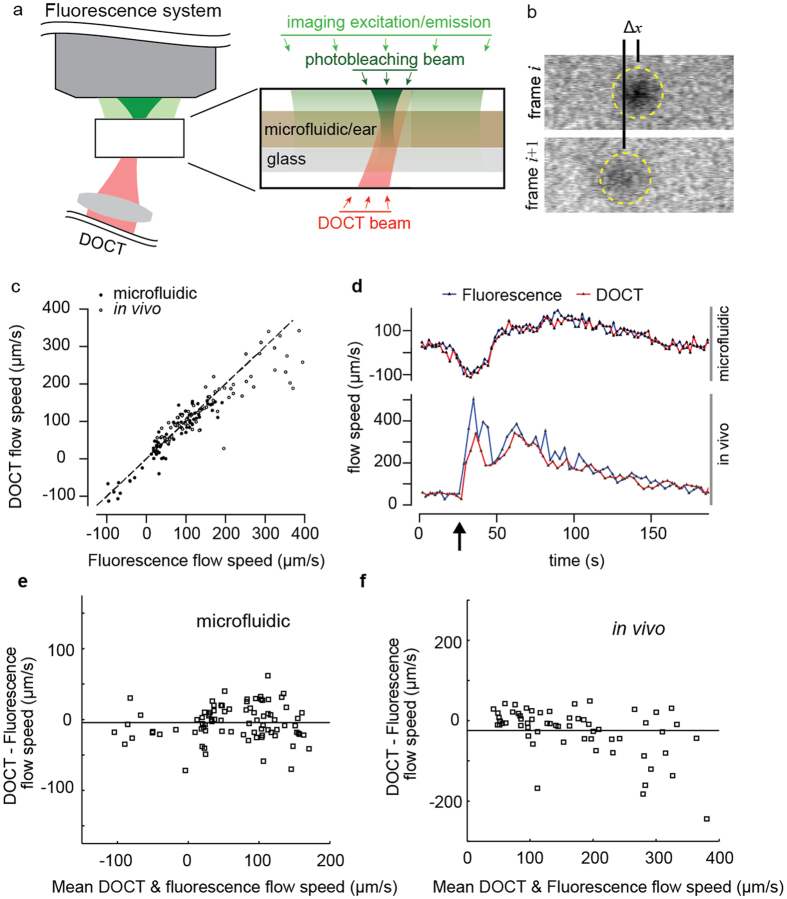
Comparison of DOCT and fluorescence photobleaching measurements of lymph proxy flow in microfluidic phantoms and *in vivo*. (**a**) Schematic of the experimental setup showing fluorescence widefield illumination (light green), focused and pulsed illumination for photobleaching (dark green) and DOCT (red). (**b**) Two cropped fluorescence frames taken from the video sequence acquired immediately after creation of a photobleached spot (in a microfluidic phantom). The translation of the spot is used to calculate flow velocity. (**c**) Comparison of simultaneous DOCT and fluorescence based flow velocity measurements in the microfluidic phantom and mouse ear. (**d**) The time-resolved velocity measurements from DOCT and fluorescence modalities in the microfluidic and *in vivo* experiments. Note that the larger discrepancies between the modalities in the *in vivo* measurements occur from 25 seconds to 65 seconds, immediately after creation of the flow bolus (arrow) when flow velocity is changing rapidly. (**e**,**f**) Bland-Altman plots, displaying the difference between DOCT and fluorescence flow measurements compared to the mean flow speed, show agreement between the two modalities in the microfluidic and *in vivo* experiments respectively.

To validate the accuracy of DOCT for measuring lymph flow velocity, we performed simultaneous measurements with DOCT and a previously described fluorescence photobleaching method^10^. A multimodal microscope was constructed that allowed imaging of a thin sample. Epifluorescence was detected from above and DOCT from below (methods, Fig. 2a). The fluorescence system combined wide-field illumination from an LED source and fixed-point photobleaching using a high-power focused laser. The photobleaching beam was modulated to create short duration (<500 ms) pulses at 3 second intervals. Wide-field fluorescence imaging was performed continuously while the photobleaching beam was off. From these images, the translational speed of the photobleached spot was calculated (methods, Fig. 2b). The DOCT beam was aligned to intersect the photobleaching beam in the fluorescence image focal plane. The angle of the DOCT beam relative to the imaging plane was 75 degrees. DOCT measurements were recorded simultaneously with fluorescence imaging. Flow velocities were calculated from the DOCT measurements as previously described in this manuscript. To create a fluorescent lymph proxy, we diluted Intralipid^®^ (20%, diluted 1:100 with water) to provide scattering and added Rhodamine-BSA for fluorescence. The scattering signal from the lymph proxy yielded a 7 dB SNR, which matched that of lymph measured from collecting lymphatic vessels *in vivo* (Supplementary Fig. 1).

We confirmed flow accuracy by imaging in microfluidic channels (320 μm width by 100 μm depth) using a pump to vary flow velocity (Fig. 2c,d). The microfluidic channel was larger than a lymph vessel and the channel walls were not as highly scattering as tissue. Thus, many of the challenges of *in vivo* lymph imaging are not recapitulated by the microfluidic phantom. To confirm flow accuracy in an *in vivo* environment, we measured flow in the lymphatic vessels of the mouse ear. The ear was selected because it was thin and compatible with our multimodal microscope that required optical access from both sides. A network of initial lymphatic vessels is present in the ear but these vessels lack the ability to pump and intrinsic flow is limited. To generate flow, a syringe needle was used to inject the lymph proxy at a site distal to a targeted lymphatic vessel. The injection pressure was adjusted to modulate the induced flow velocity. As with the microfluidic measurements, the *in vivo* flow measurements from the two modalities show high correlation (Fig. 2c,d). Additional *in vivo* measurements for validation are shown in Supplementary Fig. 4. We note that the range of induced flow velocities in both the microfluidic and *in vivo* settings (0–400 μm/s) are well matched to physiological flow speeds reported in literature^28^. These measurements provide confirmation that the DOCT method, which has been extensively validated for blood flow imaging^27^,^29^,^30^,^31^, also allows accurate measurement of lymph flow velocity.

### M-Mode DOCT Imaging of Pulsatile Lymph Flow Velocity

We prepared the afferent lymphatic vessel of the popliteal lymph node for imaging using established methods^32^,^33^. Each measurement spanned five minutes. Doppler analysis used a 0.25 sec STFT window (providing 0.25 seconds temporal resolution). A total of 67 measurements were made in 11 animals (Supplementary Table 1). Due to animal motion or pronounced fixed pattern noise that overlapped the lymphatic vessel, no flow measurements were possible in 20 of these 67 measurements. All depths within the lymphatic vessel were analyzed in the remaining 47 measurements to yield the depth and time resolved lymph flow velocity.

**Figure 3:**
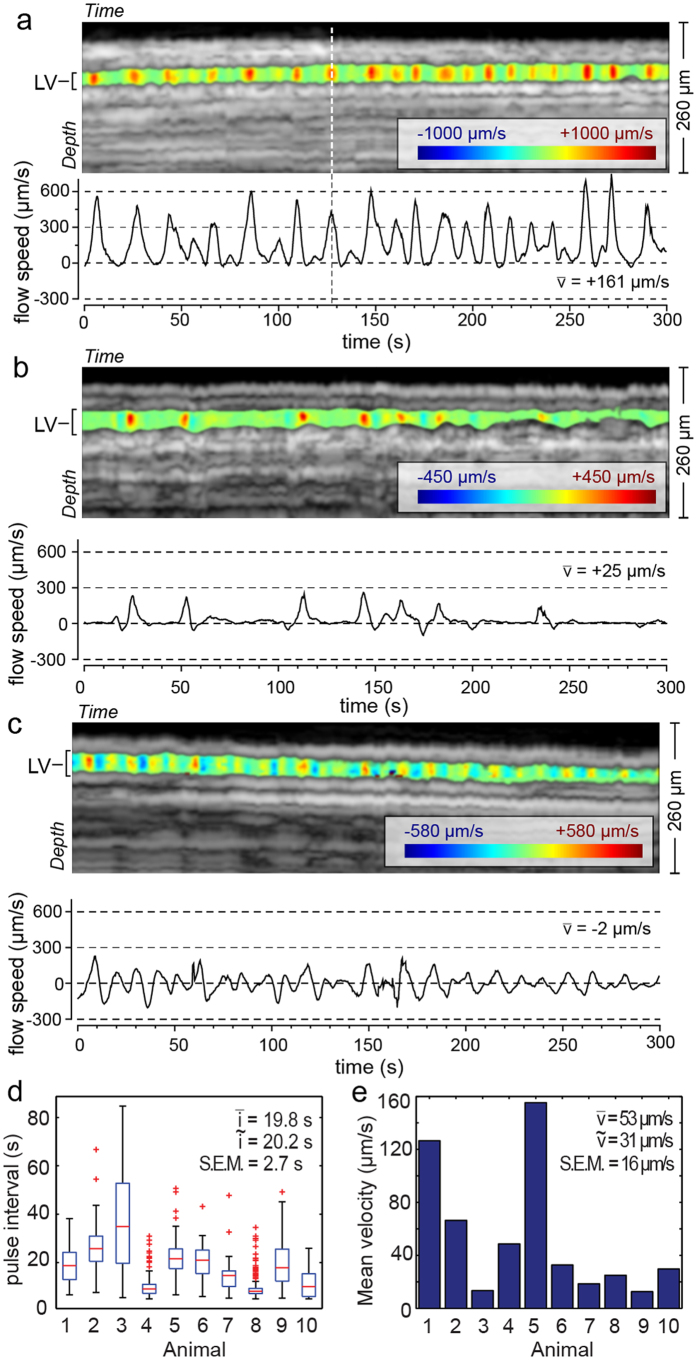
M-Mode DOCT measurement of pulsatile lymph flow velocity. (**a**) Depth and time-resolved flow velocity in the lymphatic vessel lumen overlaid on the M-Mode intensity image. The measurement location is highlighted in Fig. 1(a). The positive velocity indicates flow proximally toward the body. The animal leg was lying lower than the abdomen. The flow direction was therefore against gravity. A velocity time trace was calculated by averaging the flow velocity in the vessel over the lymphatic vessel (LV) depth. The maximum amplitude of this velocity is smaller than in the image above because of this depth averaging. (**b**) An M-Mode measurement showing periods of backflow. (**c**) A measurement in another animal showing an oscillatory flow velocity at relatively higher frequency than pulsatile flow exemplified by panel (a). (d) The average pulse interval for 10 animals showing pronounced pulsatile flow dynamics. The box plot for each animal includes data obtained over multiple five minute duration measurements. The mean pulse interval is 19.8 seconds and is much longer than respiratory or cardiac cycles. (**e**) The mean flow velocity in the same animals as in (**d**) calculated as the average over individual five minute measurements. 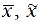 and S.E.M. denote mean, median and standard error of the mean respectively.

In most measurements (37 of 47), we observed a distinct pulsatile flow. The flow pulses were always proximally toward the popliteal lymph node. While most of these measurements (32 of 37) showed no measureable backflow (Fig. 3a), a small subset (5 of 37) exhibited transient backflow after or between pulses (Fig. 3b). In four measurements, oscillatory flow at a higher frequency was observed, but these measurements showed limited net transport (Fig. 3c). As expected, no animals showed a net lymph flow distally—independent of the elevation of the leg relative to the body (Supplementary Fig. 5). The technique showed consistent lymph flow parameters across 45 minutes in a single animal (Supplementary Fig. 6). To our knowledge, these measurements are the first direct and label-free depth-resolved measurements of pulsatile lymph flow velocities *in vivo*.

We compared the observed pulsatile lymph flow dynamics to reported values. The pulse interval (time between pulses) was extracted across the 37 measurements (10 animals) that showed strong pulsatile flow (methods, Fig. 3d). The average interval between pulses was 19.8 seconds ± 2.7 seconds (methods). Using fluorescence imaging in the axillary lymphatic vessel, Kwon *et al*. reported a flow pulse interval from 5 to 85 seconds in five animals^6^. Proulx *et al*. observed average pulse intervals of 6.3 seconds, dropping to 4.7 seconds after application of external compression to stimulate the initial lymphatic uptake of tracer^13^. Liao *et al*. also reported an interval between contractions of lymphangions of 1 to 2 seconds in young animals^32^. However, how the contraction of a single lymphangion relates to lymph flow is the subject of active research, and thus contraction and flow intervals may not agree. Transient flow velocity reversals similar to that observed by DOCT (Fig. 3b) were also reported using a cell tracking method^34^. The range of lymph flow velocities in the mouse afferent lymphatic vessel of the popliteal lymph node is not well established. However, the flow velocities measured at this site by DOCT (53 μm/s ± 16 μm/s, methods, Fig. 3e) are in reasonable agreement with relevant reports. Using fluorescence recovery after photobleaching, Bouta *et al*. reported a mean velocity value of 100 μm/s in the hindlimb^28^. In the axillary lymphatic, Kwon *et al*. measured a lymph flow velocity ranging from 280 to 1,350 μm/s, which included larger collecting lymphatic vessels as well. The faster velocities previously reported may have been affected by contrast injections to highlight the lymphatic vessels. Dixon *et al*. reported velocities of 350 to 1,500 μm/s in larger rat mesenteric lymphatic vessels using cell tracking^35^.

### DOCT-Based Measurement of Lymph Volumetric Flow Rate and Vessel Contraction

The M-Mode Doppler method described above allows flow velocity quantification at a single transverse location with high temporal resolution (0.25 seconds). However, because measurements are limited to this location, it is not possible to calculate the average lymph flow velocity within the vessel (i.e., spatial average across the vessel cross-section). In addition, the dynamic changes in the cross-sectional area of the lymphatic vessel cannot be accurately measured from M-Mode acquisitions. Without each of these parameters, it is not possible to calculate the volumetric lymph flow rates (measured for example in μL/h). To address this, we implemented a second measurement protocol based on B-Mode (frame) Doppler acquisition and analysis. In B-Mode Doppler, temporal resolution is slightly sacrificed to enable two-dimensional imaging of lymph flow in a plane approximately orthogonal to the flow direction. From these B-Mode images, both the cross-sectional area and average flow can be derived.

**Figure 4:**
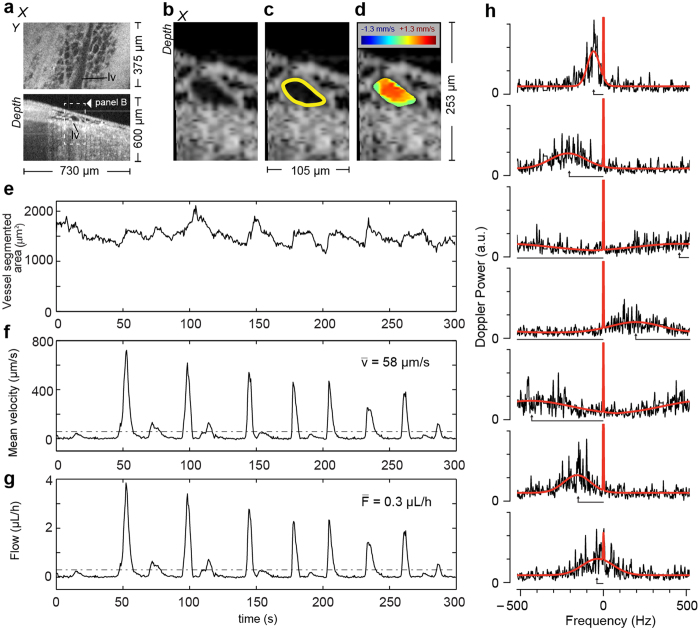
B-Mode DOCT measurement of spatially and temporally resolved lymph flow velocity, vessel cross-sectional area and volumetric flow rates. (**a**) Three-dimensional OCT datasets viewed in *en face* (upper panel, lv: lymphatic vessel) and cross-sectional (lower panel) presentations are used to identify lymphatic vessels and select the flow measurement cross-section. Representative image of the time series (Supplementary Video 1) showing the vessel cross-section (**b**), its segmentation result (**c**), yellow curve) and the calculated flow velocity spatial distribution (**d**). The velocity image was filtered with a two dimensional median filter and unwrapped. (**e**) The segmented cross-sectional area of the lymphatic vessel is reported over the five minutes measurement. (**f**) The mean velocity calculated over the vessel cross-sectional area is reported with its mean value indicated by the dashed line. (**g**) The lymphatic volumetric flow is calculated as the product of the cross-sectional area and the mean velocity (methods). The velocity measurement resolves the pulsatile flow, similar to previous measurements. Interestingly, the vessel cross-section profile shows changes in accordance with the flow pulses. (**h**) Seven spectra (black line) at a particular spatial location in the vessel during a flow pulse show high velocity frequency components being wrapped, i.e. appearing on the right side of the frequency scale. The fitting model in red operates on a circular coordinate system. The length of the black arrow pointing to the center of the broad Gaussian indicates the value of the frequency/velocity estimator after unwrapping.

In a B-Mode Doppler acquisition, the beam was rapidly scanned across the lymphatic vessel (Fig. 4a). We used a sinusoidal scan pattern at 1.04 kHz. With our 50 kHz A-line system, this provided 48 total A-lines per frame (24 in the forward scan and 24 in the backward scan). The scanned field was constrained to ~100 μm, a value chosen to be larger than the lymphatic vessel diameter. We then constructed 48 separate M-Mode measurements associated with each of the 48 A-lines in each frame. In these datasets, the sampling frequency was set by the frame rate (1.04 kHz), whereas the sampling rate in the direct M-Mode method was set by the A-line rate (50 kHz).

The 48 M-Mode datasets were analyzed separately in a manner similar to that described above. The temporal analysis window was expanded to 0.96 seconds (1000 frames). First, the average scattering magnitude was calculated to generate a time-varying anatomical image revealing the lymphatic vessel cross-section (Fig. 4b). The luminal surface of the vessel was segmented from these images (methods, Fig. 4c). Next, Doppler analysis was performed to generate two-dimensional images of flow velocity (Fig. 4d, Supplementary Video 1). From the transverse flow velocity profile and the luminal segmentation, it is straightforward to calculate the average lymph flow velocity within the vessel (Fig. 4e), the cross-sectional area of the vessel (Fig. 4f), and the volumetric lymph flow (methods, Fig. 4g). We note that the measurement of lymphatic vessel cross-sectional area can also be used to directly monitor lymphatic vessel contraction – a key parameter in the functional assessment of the lymphatic pumping system. To our knowledge, these data represent the first direct and temporally resolved measurement of lymph velocity profiles. The ability of DOCT to simultaneously measure each of these parameters (flow velocity, contraction, and volumetric flow rate) is extremely powerful in studies of lymphatic function^35^.

The B-Mode Doppler analysis algorithm was slightly modified from that used for M-Mode analysis because of the longer time separation between measurements (960 μs in B-Mode Doppler vs 20 μs in M-Mode Doppler), and the corresponding reduction in the Nyquist limit (521 Hz vs 25 kHz). Because measured Doppler shifts approach and even exceed this 521 Hz limit, the analysis approach must account for the wrapping effects in the calculated spectrum. We modified the model-fitting algorithm to operate on circular data, i.e., to wrap across the Nyquist boundary (Fig. 4h). After fitting, the detected Doppler shift must be unwrapped to remove jumps by twice the Nyquist frequency. In our studies, the detected Doppler shift only slightly exceeded the Nyquist boundary. This allowed a relatively straightforward unwrapping approach based on remapping the lower quadrant of detected Doppler frequency shifts (methods, Supplementary Fig. 7c).

## Discussion

We have demonstrated an imaging technique that allows direct measurement of lymph flow velocity *in vivo.* The method operates without need for injected labels that can alter the physiology to be measured. In addition, the high temporal resolution of the approach reveals pulsatile lymph flow dynamics. Finally, using B-Mode (frame) Doppler methods, the lymph volumetric flow rate can be measured at high temporal resolution. These capabilities address a long-standing need within the lymphatic research field, and the deployment of DOCT could catalyze studies into the regulation and dysregulation of lymph flow in health and disease. As with any new technology, the availability of instrumentation will influence the pace of adoption. The algorithms and methods for lymph flow imaging are not specific to a single OCT implementation, and thus can be adopted to operate in most existing and future systems. This combined with the growing use of OCT in preclinical and biological research may accelerate adoption of DOCT in the lymphatic research community.

OCT technologies can span a large range of spatial resolutions and imaging speeds. The system used here featured moderate resolutions of 6 μm axial and 11 μm transverse and moderate speeds of 50 kHz. Extending these methods to operate with higher resolution OCT systems offering isotropic resolution below 4 μm and speeds above 100 kHz is a promising research direction. Higher resolution will allow a more detailed visualization of lymph flow profiles, and higher speeds can be used to increase the temporal resolution of the frame method, or to simultaneously measure flow at multiple sites within a lymphatic vessel. The latter may allow studies into the coupling of neighboring lymphangions, and the role of this coupling in the generation of lymph flow. Until now, such studies rely solely on modeling^36^,^37^.

A drawback of the approach is the limited imaging depth, which required resection of the skin in the hindlimb lymphatic model. OCT has an imaging penetration of approximately 2 mm, but even within this range signals attenuate rapidly with depth. The lymphatic vessels imaged in these studies were superficial and it is not clear if the technique can translate to vessels that are more than several hundred microns below the tissue surface. This limitation is not unique to OCT as high-resolution fluorescence-based techniques also require the removal of skin^32^,^33^. Methods using near-infrared fluorescence can increase imaging depth, but are not able to image wall motion to measure lymphatic contraction without skin removal due to skin scattering^13^,^33^,^38^. Though DOCT currently requires skin removal, it advances the field of lymphatic research by enabling label-free, high-speed, and simultaneous measurements of lymphatic contraction, lymph flow velocity, and lymph volumetric flow in this experimental setting.

## Methods

### DOCT instrumentation

Hindlimb and ear/microfluidic imaging was performed with two separate but similarly designed custom-built DOCT systems. The design of the instrument used for hindlimb imaging is summarized in Supplementary Fig. 8 and described in more detail in Ref. 39. The system used a polygon mirror swept-wavelength laser source. The axial scan rate was 50 kHz, with a full optical bandwidth of 100 nm, centered at ∼1300 nm, giving a 5.3 μm axial resolution in tissue. With 10 mW on the sample, this system provides a sensitivity of ~105 dB. The beam is collimated to 2.8 mm and focused with a scan lens (LSM02, Thorlabs), giving a theoretical scanning beam spot size on the tissue in the center of the field of view of ~11 μm. Background subtraction, dispersion compensation, k-space interpolation, Fourier transformation and image generation are performed as previously described^39^. Three fix delay reflections were added to each system as reference for phase instability correction^40^,^41^,^42^,^43^. Phase instability occurs in wavelength-swept source based OCT systems because of imperfect synchronization of the acquisition system with the laser sweep. It has been shown that this results in trigger jitter which induces timing fluctuations of the interference signal and corresponding depth-dependent phase errors between A-scans. The first reflection was obtained by using a 4% beam sampler (partial reflection mirror) and a mirror in the free-space sample arm. It was placed at zero-delay to correct for depth-invariant phase shifts by subtracting the phase evolution at that depth from the entire M-scan^40^. The second reflection is obtained by sampling a portion of the sample light with a fiber coupler. This light is directed to a separate free-space path containing a microscope glassplate. The front and back surfaces of this glassplate provide the second and third calibrating signals. These signals are placed at the edge of the imaging range and used to correct for depth-dependent phase jitter. More specifically, the position of these signals relative to the zero-delay position and associated phase signals are used to calculate a linear phase term in depth in each A-scan, which is subtracted from that A-scan^40^.

The system used for validation studies was similar to that described above but had the following differences. It operated at an A-line rate of 80 kHz with a 140 nm bandwidth centered at 1285 nm, giving a ~5 μm axial resolution in tissue. The power on the sample was ~10 mW and the sensitivity around 105 dB. The microscope for this system is illustrated in Supplementary Fig. 9. This system did not include a beam scanner; the signal was measured at a single location with a spot size of ~23 μm.

### Animal preparation

For fluorescence photobleaching/DOCT experiments using the multimodal microscope (Fig. 2), 8–12 week old nude female mice (28–32 g) were used. Nude mice were selected to eliminate hair from the imaging field. The mice were anesthetized using a Ketamine/Xylazine 90 mg/9 mg per kg body weight. After confirmation of anesthetic plane, mice were placed in prone position with the ventral side of the right ear on a 1 cm elevated translucent glass cover slip. The edge of the ear was circumferentially held in position using ½ inch wide tape. A small drop of water under the ventral side of the ear adhered the ear to the cover glass. A 30 ½ gauge needle connected to PE10 Polyethylene Tubing and a ½ cc U-100 28 ½ gauge insulin syringe was carefully inserted between skin layers in the tip of the ear. The insulin syringe was attached to a syringe pump (Pump11 Elite, Harvard Apparatus) to control injection speed. A Rhodamine-BSA (Invitrogen Catalog #A23016)/0.2% Intralipid^®^ (Fresenius Kabi, 20% diluted 1:100 with water) 1:1 mixture was injected at 5 μL/min for 2 seconds to generate flow in the lymphatic vessels.

For DOCT experiments in the hindlimb (Figs 3 and 4), 8–16 week old C3H male mice (26–36 g) were used. Again, mice were anesthetized using a Ketamine/Xylazine 90 mg/9 mg per Kg body weight. The surgical procedure was performed as described in Liao *et al. PNAS*^32^ and Liao *et al*. *Journal of Biological Methods*^33^. Exposed tissue was kept hydrated using physiological saline. Mice were euthanized at the conclusion of the imaging experiment. The animal experiment protocol was reviewed and approved by Institutional Animal Care and Use Committee of the Massachusetts General Hospital. The procedures were performed in accordance with the approved guidelines.

### Data analysis

The interval between pulses was measured from the depth averaged velocity vs time profiles. Peaks were located by the Matlab findpeaks function with a minimum peak height larger than one third of the mean of the five largest peak amplitudes. The boxplots were calculated with the Matlab function of this name. The central red line indicates the median, while the lower and upper edges of the box are the 25th and 75th percentiles respectively. When shown, the whiskers extend to the lowest and highest datum still within 1.5 the interquartile range of respectively the lower and the upper quartile. Outliers are plotted as individual red crosses if they are not included between the whiskers.

The average interval and average mean velocity were calculated by first averaging the measurements taken in each single animal and then averaging over the group by animal. The error reported is the standard error of the mean (S.E.M.) calculated based on the animal to animal variability.

### DOCT hindlimb imaging procedure

The mouse was placed on a tilted stage (15 degrees) to generate a Doppler angle (Supplementary Fig. 3a). A primary artery/vein pair was visible by eye and was used to locate the likely position of the lymphatic vessel (runs approximately parallel to the blood vessels). The animal was positioned such that these vessels align to the direction of maximum slope. The stage was then mechanically attached to the optical table, oriented such as having the maximum slope perpendicular to the fast axis scanning direction (Supplementary Fig. 3a). Real-time OCT cross-sectional structural images (fast axis scanning, slow axis amplitude set to zero) were used to locate the lymphatic vessels on either side of the blood vessel. The image reconstruction was performed to generate isotropic images (same transverse and depth scale factors). This simplified discrimination of approximately round lymphatic vessels from other low-scattering structures such as fat cells. By moving the imaged frame along the slow-axis direction, the continuity of the presumed lymphatic vessel was confirmed. This also allowed confirmation that the lymphatic vessel runs along the direction of maximal slope. If not, another location can be chosen or the stage can be rotated. Next, the lymphatic vessel of interest was laterally centered in the field of view by motion of a translation stage. Finally, a three-dimensional volume spanning a 1.4 × 1.4 mm scanning area (464 A-Scans per B-Scans, 464 B-Scans) was acquired (Supplementary Video 2). This volume was then analyzed in ImageJ (NIH) to confirm the position and orientation of the lymphatic vessel. This three-dimensional dataset was also used to later measure the Doppler angle (see section Calculation of Doppler angle).

### DOCT/fluorescence imaging procedure

The fluorescence system is a slightly modified version of a previous experiment al system^10^. The schematic representation of the experimental setup is shown in Supplementary Fig. 9. Fluorescence images were obtained with a 20x 0.40 NA objective (LD Achroplan, Zeiss) mounted in an epifluorescence microscope (Axioskop, Zeiss). Wide-field illumination was performed through the epi-illumination port of the microscope by a green collimated LED (M530L3-C4, Thorlabs). The filter-set was a standard combination for TRITC/CY3.5. The images were acquired with an intensified charge-coupled device videocamera (C2400–68, Hamamatsu Photonics) and digitized with a USB frame grabber (USB-AVCPT, Sabrent) at 30 frames/s. A CW 532 nm laser (Verdi, Coherent) combined with a fast electronic shutter (LS6Z2, Uniblitz) were used to induce photobleaching. The laser path was overlapped on the LED illumination by using a beam-splitter, and aligned to appear in the center of the field of view. A second shutter was placed before the camera to prevent over exposure during photobleaching. The operation of the shutters was controlled with a USB interface card (NI USB 6259, National Instruments) driven by Labview (National Instruments). The laser power on the sample was ~50 mW. The shutter was open to the laser for durations of ~100 ms and ~500 ms every ~3 seconds for the *in vivo* and *in vitro* experiment respectively. The DOCT sample beam illuminated the sample from the bottom side of the microscope. The light was collimated, focused and reflected on the hypotenuse of a right angle prism to introduce a 75 degree Doppler angle. For alignment purposes, red light was coupled into the system to center the DOCT spot on the field of view to overlap with the photobleaching spot. The DOCT beam and the direction of motion define the z-x plane. The x direction corresponds to horizontal lines on the camera image. M-Scan acquisition and frame video recording were started manually approximately at the same time. Each sequence of frames following a laser illumination was processed individually to obtain a single velocity value. The first five frames after the shutter opening were used to estimate the velocity. After these frames, the photobleached spot had either translated out of the field of view, or had faded due to diffusion. Background fluorescence frames were calculated using 30 frames acquired during the time-period after the photobleached spot had disappeared. This background frame was subtracted from each of the five analyzed fluorescence images to enhance contrast for the photobleached spot. Each image was later vertically averaged along the direction perpendicular to the flow velocity to obtain an intensity profile. The minimum of that profile, which corresponds approximately to the photobleached spot centroid, was located fitting the measured profile to a 11^th^ order polynomial curve and finding the minimum of that curve. The location of the minimum over the five frames was fit to a linear curve. The velocity was extracted as the slope of this fitted line.

### M-Mode and B-Mode Doppler processing

Detailed steps involved in the Doppler analysis of M-Mode (Fig. 3) and B-Mode (Fig. 4) datasets are presented in Supplementary Notes 1 and 2, respectively.

### Calculation of Doppler angle

The Doppler angle was measured using the three-dimensional dataset acquired prior to each flow velocity measurement (see DOCT hindlimb imaging procedure). A single cross-sectional image aligned to the longitudinal axis of the lymphatic vessel was generated (Supplementary Fig. 3a,b). Because the lymphatic vessel does not necessarily align parallel to the tissue surface, it was necessary to measure the vessel angle directly including the influence of refraction at the air/tissue boundary. Within this image, we draw two right angle triangles aligned to the tissue and vessel boundaries (Supplementary Fig. 3b). Each triangle is drawn with the same transverse extent Δ. Next, the physical distances (e.g., measured in μm by multiplying the number of pixels by the scale factor) for the transverse and depth sides (Δ*z*_*a*_ and Δ*z*_*t*_) of these two triangles are measured. We note that the depth distance must take into account the different scale factor (e.g., μm/pixel) in the depth dimension for air (*n* = 1) and tissue (*n*_*t*_ = 1.38) spaces. These distances are illustrated on Supplementary Fig. 3b. The Doppler angle can then be calculated from these parameters and the index of refraction of tissue, *n*_*t*_, as:


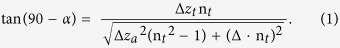


## Additional Information

**How to cite this article**: Blatter, C. *et al*. *In vivo* label-free measurement of lymph flow velocity and volumetric flow rates using Doppler optical coherence tomography. *Sci. Rep.* **6**, 29035; doi: 10.1038/srep29035 (2016).

## Supplementary Material

Supplementary Information

Supplementary Video 1

Supplementary Video 2
