# Supplementary material for: In vivo label-free measurement of lymph flow velocity and volumetric flow rates using Doppler optical coherence tomography
Source: Sci Rep. 2016 Jul 5;6:29035. doi: 10.1038/srep29035 (PMC4932526; doi:10.1038/srep29035)
Supplement: Supplementary Information [file srep29035-s1.pdf]

In vivo label-free measurement of lymph flow velocity and volumetric flow rates using Doppler optical coherence tomography

Cedric Blatter[1,3], Eelco F.J. Meijer[2,3], Ahhyun S. Nam[1,3], Dennis Jones[2,3], Brett Bouma[1,3], Timothy P. Padera[2,3,§], and Benjamin J. Vakoc[1,2, §,*]

[1]Wellman Center for Photomedicine, Massachusetts General Hospital, Boston, Massachusetts 02114; [2]Edwin L. Steele Laboratories for Tumor Biology, Department of Radiation Oncology, Massachusetts General Hospital Cancer Center, Boston, Massachusetts 02114; and [3]Harvard Medical School, Boston, Massachusetts 02115

[§]Authors contributed equally. *Correspondence and requests for materials should be addressed to B.J.V. (email: bvakoc@mgh.harvard.edu)

Supplementary Figures

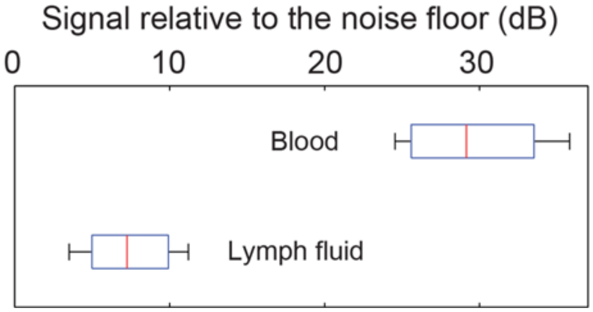

Supplementary Figure 1. The signal to noise ratio (SNR) of lymph and blood scattering signals in the mouse hindlimb. The lymph signal was calculated as the average signal in the middle of a lymph vessel across 12,000 A-lines. Averaging was performed across signal powers, i.e., prior to log-scaling. The noise was calculated similarly in a region of air. The signal for blood was measured from cross-sectional images selecting superficial vessels and averaging across the interior of the vessel. An average SNR for lymph and blood was calculated for each animal. The variability across animals is represented by the boxplot display (methods).

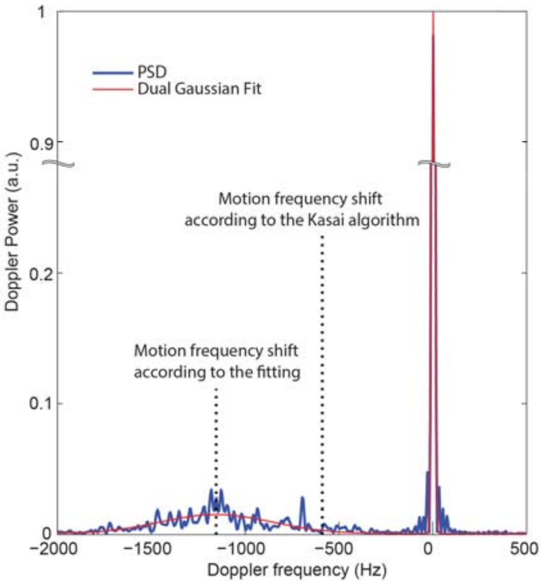

Supplementary Figure 2. A comparison of the Doppler shifts detected by an existing algorithm (Kasai method[8,9]) and the proposed Doppler technique when applied to the same lymph measurement data of 12,000 consecutive A-lines (50 kHz A-line rate). The Kasai approach analyses the complete signal and cannot discriminate between the lymph and static components. As such, it reports a Doppler shift that is intermediate to the lymph and static components. The proposed method employs a dual Gaussian fitting (red line) of the power spectral density (PSD) which allows the two components to be separated. The center frequency of the broad Gaussian is used to estimate the lymph flow induced Doppler shift. Note that the y-axis was broken for a better visualization of the lymph component.

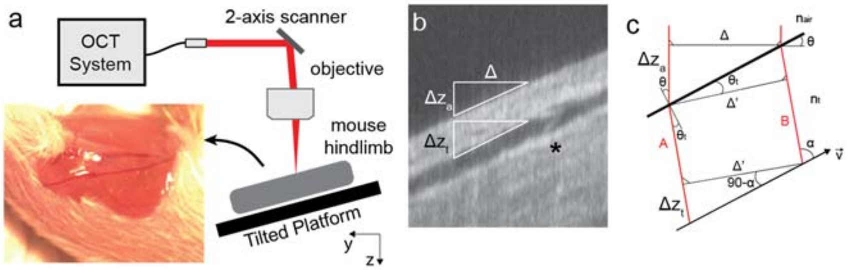

Supplementary Figure 3. Hindlimb imaging stage and calculation of the Doppler angle from a three-dimensional OCT dataset. (**a**) The mouse is held on a tilted platform that can be positioned and rotated under the imaging microscope. The angle between the beam and titled platform was set to 75 deg to be sufficiently far from 90 degrees to give accurate measurements while allowing all imaged animals to fit underneath the objective. For lymphatic vessel oriented parallel to the platform, a 1 degree error in the Doppler angle measurement yields an error smaller than 10% of calculated velocity. This level of error is significantly smaller than the physiological variations in flow across time and across animals and is therefore acceptable in this experimental context. (**b**) A cross-sectional image aligned to the longitudinal axis of the lymphatic vessel and extracted from the three-dimensional dataset. The asterisk * notes the visibility of a lymphatic valve in the OCT image. Two right angle triangles have their hypotenuse aligned to the tissue and vessel boundaries respectively, with Δ the transverse extent in air, Δz_a and Δz_t the depth physical distances in air and tissue respectively. Those values are measured and employed to calculate the Doppler angle. (**c**) A geometric illustration of the OCT imaging beams including the effect of refraction at the air/tissue interface used in the derivation of the Doppler angle expression presented in Supplementary Note 3.

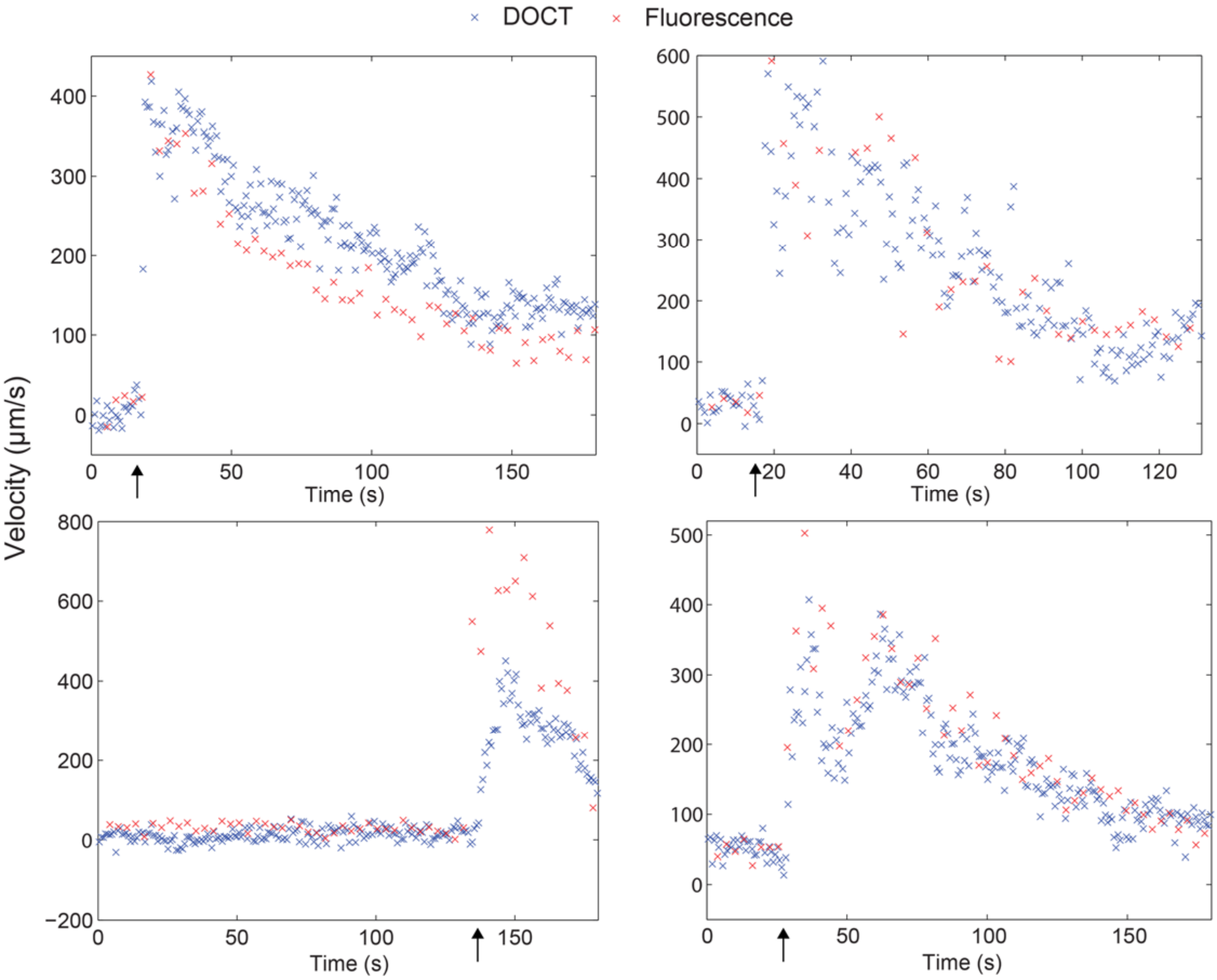

Supplementary Figure 4. Four additional *in vivo* measurements of lymph proxy flow velocity in a perfused lymphatic vessel in a nude mouse ear acquired simultaneously with DOCT (blue cross) and fluorescence photobleaching (red cross). The flow was generated by a syringe pump. The arrow indicates the approximate time of the creation of a flow bolus. The scale factor error in the lower left panel is attributed to an error in the Doppler angle. Instrumentation constraints prevented three-dimensional OCT imaging in this multimodal setup. This prevented direct measurement of the Doppler angle in each sample/measurement. Instead, the Doppler angle was assumed to match that of the glass surface on which the ear was placed. Variations from this nominal angle can affect the scale factor of the DOCT measurements (but not the temporal shape). *In vivo* measurements in the hindlimb utilize direct measurement of Doppler angle and are not affected by this uncertainty.

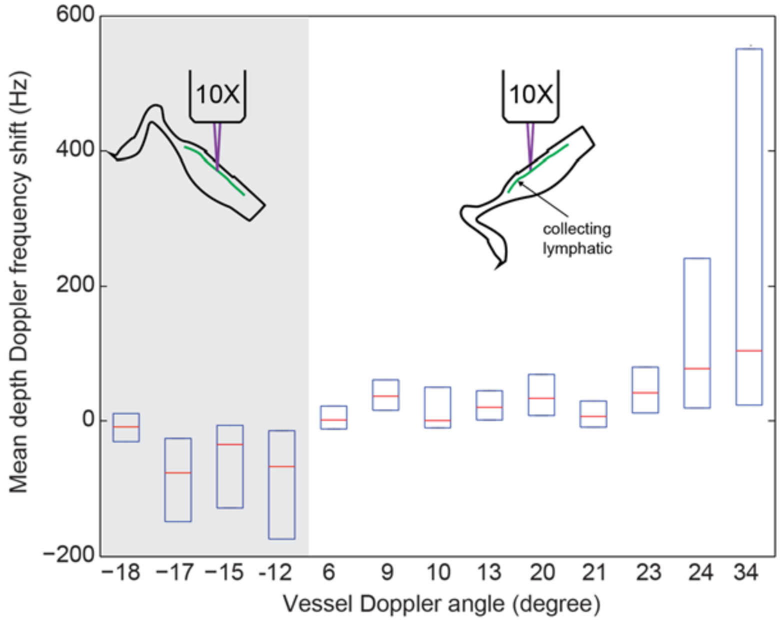

Supplementary Figure 5. The direction of flow was always measured to be toward the popliteal lymph node, independent of the elevation of the leg relative to the body. Depth-averaged M-Mode Doppler shifts across 37 measurements and 10 animals were binned according to the measured Doppler angle (rounded to the nearest integer). The detected Doppler shifts across time and across measurements for each bin were then represented using a boxplot (methods). These data demonstrate that the sign of the measured Doppler shift reverses as the gross elevation of the leg (relative to the body) is reversed. This confirms that the measured Doppler shift indicates flow toward the lymph node as is expected by physiology. We note that while the gross elevation and the lymphatic vessel angle are correlated parameters, they are not identical. Because of this, these data should not be used to define a biological relationship between limb elevation (or gravitational force) and resulting lymph flow, although this measurement technique does allow for this relationship to be studied in the future.

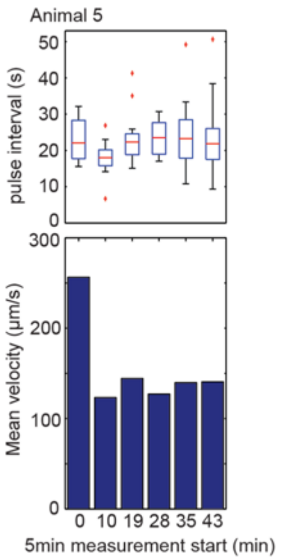

Supplementary Figure 6. Reproducibility measurement of flow pulse intervals (time between pulses) and mean flow velocity in the afferent lymphatic vessel of the popliteal lymph node in one mouse (animal 5), at the same location over time. In the upper diagram, the pulse interval is represented by boxplots for 6 measurements of five minutes each (methods). In the bottom plot, the mean velocity is represented for the same measurements.

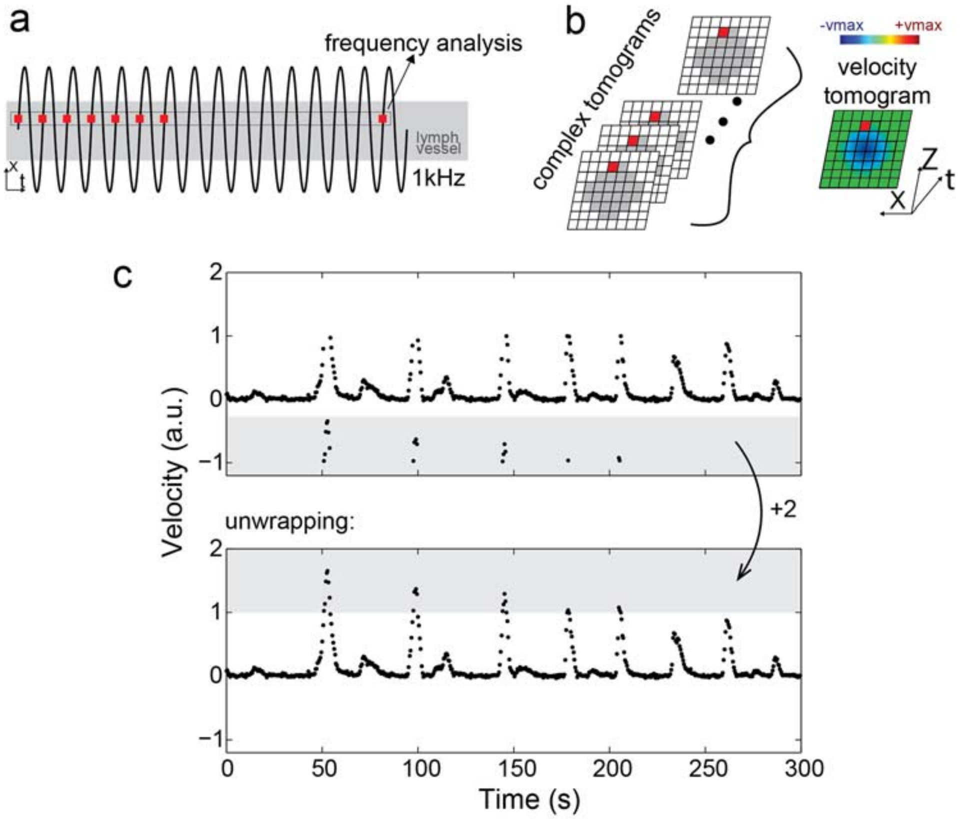

Supplementary Figure 7. An illustration of Doppler analysis from B-Mode datasets. (**a**) Fast scanning (x) operation over a lymphatic vessel shown from a top *en face* view. A time series of 1000 A-lines (marked red) is selected for frequency analysis to extract a Doppler frequency. (**b**) This process is repeated at each depth and each lateral location over the *complex* (phase and amplitude value) dataset to obtain a velocity tomogram shown on the right with a velocity color map. (**c**) Velocity time profile at one lateral location and one depth showing that high flow velocity values during flow pulses can exceed the Nyquist detection limit and induce wrapping effects. Here, the velocity range was normalized to [-1,1] where +/- 1 represents +/-Nyquist. Unwrapping was performed assuming flow measurements in the range of -1 to -0.35 were wrapped, and adding 2 to these values (lower panel). This approach works well for the small degree of wrapping observed in our system. Larger wrapping may require more complex 2D unwrapping algorithms.

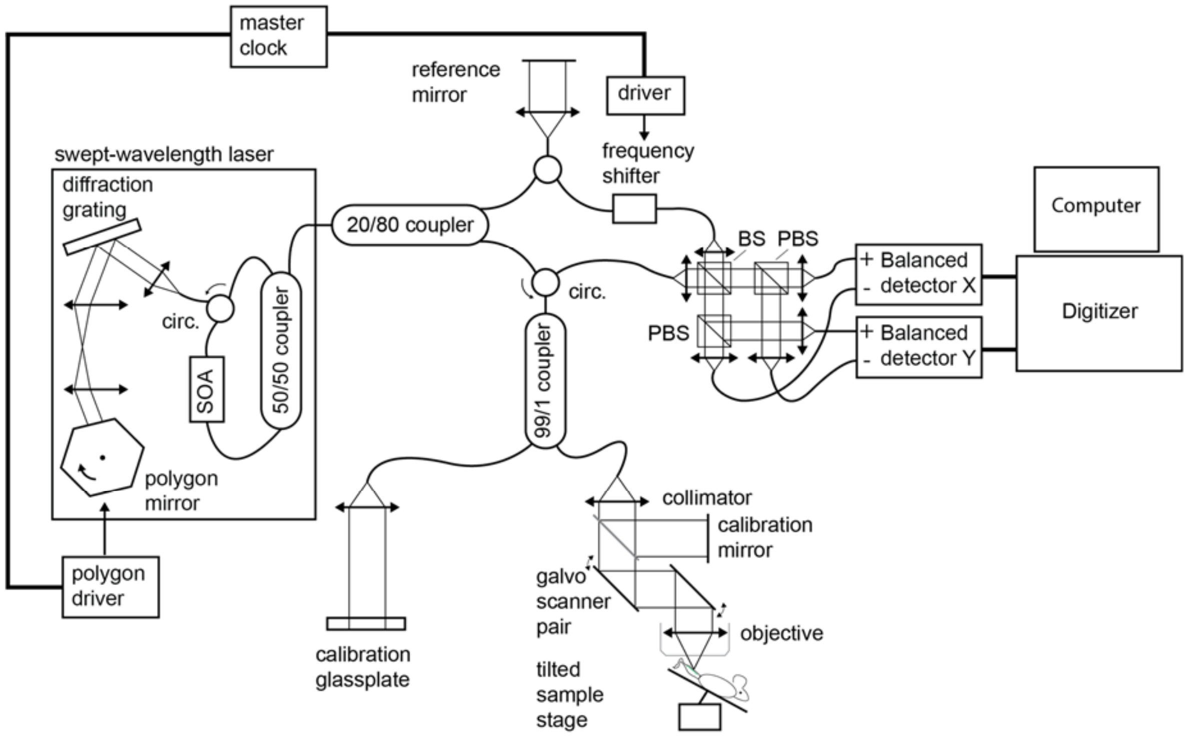

Supplementary Figure 8. Schematic of the 50 kHz DOCT system used for hindlimb lymph flow velocity measurement. The system employs a swept-wavelength laser source based on a polygon mirror (Lincoln Laser) and a semiconductor optical amplifier (SOA) inside a fiber ring cavity. A 10/90% coupler is used to split the laser output between reference and sample arm. The sample light is further split with a first fiber coupler; 1% of the light is collimated and reflected on a glass plate, which is used as a calibration signal for phase jitter correction[1]. Most of the light is directed to a free space microscope. After collimation, a beam sampler (partial reflection mirror) is used to direct 4% of the light to a mirror to generate a calibrating signal. A pair of galvo-scanners is used to steer the beam for three-dimensional imaging. The galvanometer-based optical scanners are not resonant devices. An acousto-optic frequency shifter (Brimrose) operating at 25 MHz is used to remove the depth degeneracy. Polarization-diverse and balanced detection are achieved with two polarizing (PBS) and one non-polarizing (BS) free-space beamsplitters and two balanced receivers (New Focus). Although both polarisation channels are available for processing, only one of the channel was actually used. The electrical signals are digitized at 100 MS/s (Signatec) and the data transferred to the computer. A master clock locks the phase of the polygon driver and the frequency shifter. The 80 kHz system was nearly identical in design to the 50 kHz system but used a faster polygon scanning mirror (Copal electronics) and different balanced receivers (Thorlabs). The sample arm schematic is visible in Supplementary Fig. 9.

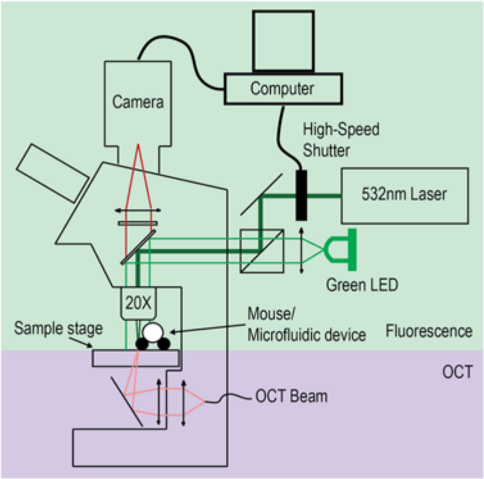

Supplementary Figure 9. Schematic of the multimodal DOCT/fluorescence system used for flow validation. The upper green area depicts the fluorescence imaging system and the lower purple depicts the sample arm of the DOCT system. The DOCT beam was tilted by 75 deg relative to the microscope imaging platform. The angle was verified by geometric measurement of the beam translation across distances of several tens of centimeters.

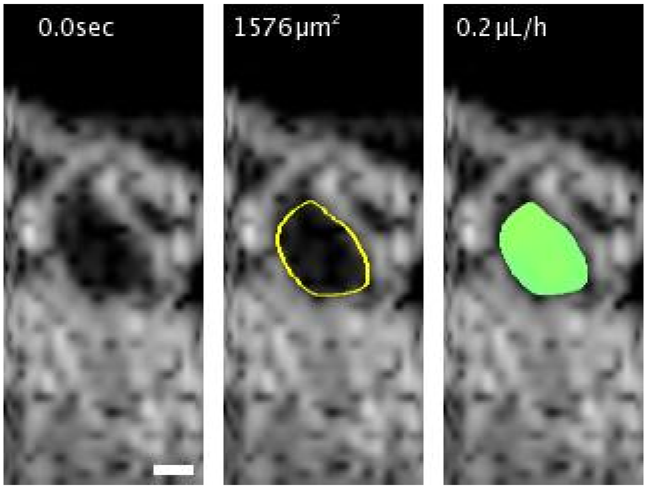

Supplementary Video 1. The simultaneous measurement of spatially and temporally resolved lymph flow velocity, vessel cross-sectional area and volumetric flow rates by B-Mode DOCT. Left: vessel cross-section. The time is displayed at the top of the panel. Middle: vessel cross-section segmentation result (yellow curve) and its area indicated in μm^2. Right: calculated flow velocity spatial distribution displayed using the velocity magnitude colorbar of Fig. 4. The volumetric flow rate is indicated at the top. Scale bar denotes 20 μm.

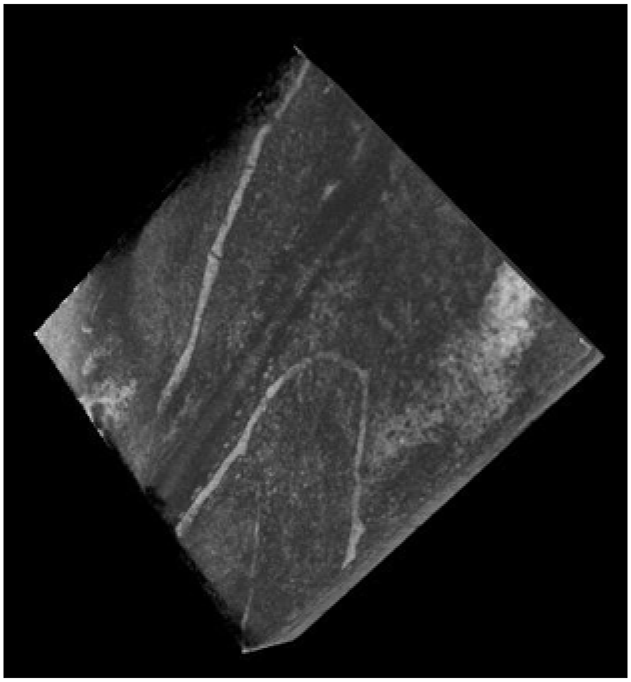

Supplementary Video 2. Volume rendering of a three-dimensional dataset similar to those used to confirm the position and orientation of the lymphatic vessel and to measure the Doppler angle. The displayed volume spans a 2 x 2 mm scanning area (464 A-Scans per B-Scans, 464 B-Scans). High scattering signals are represented by black values. Lymphatic vessels appear as white (low scattering) structures.

Supplementary Table 1. Summary of M-Mode Doppler flow measurements. 67 M-Mode measurements on 11 different animals are reported below. The location column indicates if the measurement in a given animal was repeated at the same location (same letter) or performed at a new location. The mean interval was not calculated on data showing no or low pulsatility and having poor structural M-Mode image quality. Up and down leg orientations indicate the elevation of the leg relative to the body (see Supplementary Fig. 5). Measurements #2, 9, 53 and 6 are presented in Fig. 3a, b, c and Fig. 4 respectively.

Measurement #	Mouse	Location	Comment	Mean interval (s)	Leg orientation
1	1	A	Location moved after first half	25.8	up
2		B	OK	15.77	down
3		C	OK	23.84	down
4	2	D	OK	29.83	up
5		E	OK	21.6	up
6		F	OK	24.14	down
7		G	OK	53.2	down
8	3	H	OK	35.08	up
9		H	OK	36.84	up
10		H	OK	76.2	up
11		H	Fix pattern noise in the vessel	N/A	up
12		H	OK	27.6	up
13		H	OK	40.12	up
14	4	I	Fix pattern noise in the vessel	N/A	down
15		I	OK	9.45	down
16		I	OK	7.67	down
17		I	OK	10.89	down
18		I	OK	10.85	down
19		I	OK	14.48	down
20	5	J	OK	23.06	down
21		J	OK	18.18	down
22		J	OK	23.54	down
23		J	OK	23.52	down
24		J	OK	24.89	down
25		J	OK	24.35	down
26	6	K	High reflection in the M-Scan, pulses visible	N/A	down
27		K	High reflection in the M-Scan, pulses visible	N/A	down
28		K	Fix pattern noise in the M-Scan, pulses visible	N/A	down
29		K	High reflection in the M-Scan, pulses visible	N/A	down
30		K	Fix pattern noise in the M-Scan, pulses visible	N/A	down
31		K	Fix pattern noise in the M-Scan, pulses visible	N/A	down
32	7	L	Location moved after 2/3rd	21.75	down
33		L	OK	23.62	down
34		L	High reflection in the M-Scan, pulses visible	N/A	down
35		L	OK	17.76	down
36		L	Almost constant flow	N/A	down
37		L	High reflection in the M-Scan, pulses visible	N/A	down
38	8	M	OK	16.67	down
39		M	Location moved early	N/A	down
40		M	Wrong location	N/A	down
41		M	Wrong location	N/A	down
42		M	Wrong location	N/A	down
43		M	Wrong location	N/A	down
44		M	Wrong location	N/A	down
45	9	N	Oscillations	N/A	down
46		N	OK	10.8	down
47		N	OK	11.38	down
48		N	OK	8.21	down
49		N	OK	9.89	down
50		N	OK	9.87	down
51	10	O	Location moved early	N/A	down
52		O	OK	16.98	down
53		O	Oscillations	N/A	down
54		O	Structure in the lymph vessel	N/A	down
55		O	Location moved	N/A	down
56		P	No flow	N/A	down
57		P	Oscillations	N/A	down
58		P	Oscillations	N/A	down
59		P	Location moved after 2/3rd, pulses visible	24.9	down
60		P	OK	22.67	down
61		P	OK	24.27	down

62	11	Q	Almost no flow, constant	N/A	down
63		R	OK	11.98	down
64		R	Almost no flow, constant	N/A	down
65		R	Almost no flow, constant	N/A	down
66		R	Almost no flow, constant	N/A	down
67		R	Fix pattern noise in the vessel	N/A	down

Supplementary Note 1

M-Mode Doppler processing. To analyze M-Mode acquisitions, the acquired dataset is first processed according to previously described OCT steps to obtain a complex depth-resolved scattering profiles, or A-Scans. These A-Scans describe the depth resolved reflectivity of the sample across time. The set of all A-Scans is labeled as an M-Scan. The M-Scan is grouped into blocks of 12,000 A-Scans. Each block is analyzed to yield a Doppler shift at each depth location within the lymphatic vessel. After each block is processed, a depth-resolved velocity over time is obtained. In the first block, a static region of tissue is manually selected to be used later as a reference for bulk motion correction. For each block, the following steps are performed:

1. The A-Scans phase instability is corrected using calibration signals and existing methods[1].

2. The bulk motion is removed from the dataset. The depth with maximum signal amplitude within the previously defined static tissue region is used to calculate the phase evolution due to bulk motion, and this phase is compensated at all depths in the lymphatic vessel. This approach is effective for small magnitude axial motion. For larger motion or transverse motion on the scale of the imaging resolution, Doppler imaging can be hindered. While we did not observe this magnitude of motion in these experimental models, this level of motion may need to be overcome if the approach is translated to other models, sites, or organisms.

3. The velocity is estimated by a frequency analysis of the complex signal in a similar fashion to the joint spectral and time domain method[2]. The following processing is applied for each depth:

A. A temporal power spectral density (PSD) is estimated using Welch's method. It results from the calculation of the average squared module of the fast Fourier transform (FFT) of 6 temporal Gaussian windows, each composed of 2,400 samples, spanning the block duration (12,000 samples) with 20% overlap. A 4 time zero-padding is used for the calculation of each FFT.

B. The PSD in the frequency region of -2 to 2-kHz bandwidth, which encompasses the flow velocity encountered in the mouse hindlimb at our selected Doppler angle, is selected for further processing. Fixed pattern noise occurring at specific frequencies is removed by interpolation. In our case, these noise terms resulted from the periodicity in the polygon mirror rotation. The white noise background is then subtracted from the PSD. Finally, the PSD (without background) is fit by two unconstrained Gaussians.

$$g = a_1 e^{-\left(\frac{f-b_1}{c_1}\right)^2} + a_2 e^{-\left(\frac{f-b_2}{c_2}\right)^2}. \quad (1)$$

The first Gaussian accounts for static signal that can be measured in the lymphatic vessel because of the PSF axial extension from the surrounding tissue. The second Gaussian function represents motion. It is broader than the first because of decorrelation by lateral motion and diffusion, and it is shifted from DC (zero frequency) due to axial motion (flow). The center frequency of the second Gaussian (b_2) was selected as the Doppler frequency caused by the axial motion of lymph.

C. The Doppler frequency can be related to the axial velocity v_a, component of the motion along the OCT beam by knowledge of the swept source center wavelength λ_c and lymph group refractive index n_{lymph}:

$$v_a(z,t) = \frac{b_2 \lambda_c}{2 \cdot n_{lymph}}. \quad (2)$$

The value of n_{lymph} was assumed to be 1.33, equal to that of plasma[3].

D. The magnitude of velocity vector is calculated by knowledge of the Doppler angle of the lymphatic vessel α:

$$v = \frac{v_a}{\cos(\alpha)} = \frac{v_a}{\sin(90-\alpha)}. \quad (3)$$

A structural M-Scan is calculated by amplitude averaging the 12,000 A-Scans within each block of data (Fig. 1b). Small amplitude motion of the reference arm can happen during the M-Scan acquisition. The position of one of the calibration signals was monitored and the axial location of each velocity depth profile corrected according to this signal. The lymphatic vessel lumen is segmented based on region growing using the large intensity contrast of the lymph relative to tissue. A supervised semi-automatic algorithm locates the vessel lumen by manual specification of starting points within the vessel and thresholding based on intensity. The delineation is used to calculate a temporal velocity profile by averaging the velocity values over the segmented depth range (Fig. 3). The structural M-Scan and depth-resolved velocity time evolution are combined based on the same segmentation (Fig. 3).

Supplementary Note 2

B-Mode Doppler processing. The analysis of B-Mode (frame Doppler) datasets was similar to that of M-Mode, but modified to account for the lower sampling rate of the analyzed signals and the potential for wrapping across the Nyquist boundary. The lymphatic vessel is first located and centered within the field of view at the location of interest. Next, a sinusoidal waveform with a period of 48 A-Scans (~1kHz with the system A-Scan frequency) is used to drive the fast axis galvo-scanner (Cambridge Technology), while the amplitude of the slow axis scanner is set to zero (Supplementary Fig. 7a). The signal is recorded for about five minutes and standard OCT processing is used to obtain complex depth-resolved scattering profiles, i.e., A-Scans. The processing is applied on blocks of 48,000 successive temporal A-Scans comprising 1000 complete frames. The analysis is continued by shifting the temporal block by one half of the block duration (to provide overlap) and repeating. Over a five minute measurement, this yields 624 velocity images (Supplementary Fig. 7b).

1. The A-Scans phase instability is corrected using calibration signals and existing methods[1].

2. The block of data is reshaped to a three dimensional data set (*z*-*x*-*t*). This allows analysis of the complex signal over time for each lateral position. The processing steps to extract the velocity at each lateral position and each depth are similar to the one applied to the M-Scan.

A. The 1000 temporal points are split into 5 overlapping windows of 250 points each. The square absolute of the signal's FFT is calculated using 4 time zero-padding for each window and then averaged to obtain an estimate of the PSD.

B. Similarly to the M-Scan processing, the potential fixed noise patterns appearing at discrete frequencies are removed with interpolation (in our case at one discrete frequency).

C. Because of the reduced sampling frequency, the whole frequency range can contain frequency shifted components of interest and sometimes wrapped signals. The function fitted on the PSD is therefore composed of two constrained circular Gaussians:

$$g = a_1 e^{-\left(\frac{mod[f-b_1+1,2]+1}{c_1}\right)^2} + a_2 e^{-\left(\frac{mod[f-b_2-1,2]+1}{c_2}\right)^2} + d, \quad (4)$$

where mod is the modulo function and a frequency range of f normalized to [-1,1]. To improve the fitting accuracy, starting points are given and mostly calculated based on the PSD:

$$\begin{gathered} a_1 = abs\big(\max(PSD) - mean(PSD)\big) \\ b_1 = 0 \\ c_1 = \sqrt{0.00002} \\ a_2 = std(PSD) \\ b_2 = \xi \\ c_2 = \sqrt{0.1} \\ d = mean(PSD)\,. \end{gathered} \quad (5)$$

The starting point value ξ of the frequency shifted Gaussian is estimated by a circular complex averaging of the PSD, similarly to the algorithm proposed by Walter *et al.*[4]. The low frequency components of the PSD representing static tissue are first set to 0:

$$\begin{array}{c} P(f) = PSD(f),\ \ abs(f) > 10Hz \\ P(f) = 0,\ \ otherwise \end{array} . \tag{6}$$

ξ is then given by:

$$\xi = \angle\left(\sum_f P(f) \cdot e^{jf\pi}\right). \tag{7}$$

In addition, the range of the parameters is constrained:

$$\begin{array}{c} 0 < a_1 < 2 * \max(PSD) \\ -0.001 < b_1 < 0.001 \\ sqrt(0.000005) < c_1 < sqrt(0.00004) \\ 0 < a_2 < 2 * \max(PSD) \\ -Inf < b_2 < Inf \\ sqrt(0.01) < c_2 < sqrt(0.5) \\ 0 < d < 2 * mean(PSD) \end{array} \tag{8}$$

The frequency center of the second Gaussian (b_2) was selected as the Doppler frequency caused by the axial motion of lymph.

3. The velocity frame (*z*-*x*) is filtered with a two dimensional *complex* median filter on the velocity range scaled to [-π,π] to be robust to wrapping artifacts. This one wrap artefact was corrected by adding twice the Nyquist limit to velocities in the most 65% negative values (Supplementary Fig. 7c).

Structural images across 624 frames are obtained by amplitude averaging across the first ten (of 1000) intensity frames in each temporal block. The velocity and the structural frames series are interpolated to remove the lateral distortion caused by the sinusoidal scanning. The first frame of the series is used to find the lateral phase offset of the sinusoidal drive signal. Only the forward scan of the sinusoidal waveform was used. A similar analysis on the backward scan can be performed to improve noise performance. The intensity frames series is later registered using ImageJ plugin StackReg[5] (with rigid body constraints) to remove bulk motion over the five minutes recording. The result of the registration is used to transform the velocity image series. The lymphatic vessel is segmented using the intensity frame series and a 2D snake algorithm[6]. The vessel cross-section area is calculated as the sum of pixels within that segmentation and scaled according to the depth and lateral scales. The mean velocity is calculated as the angle of the complex sum of imaginary exponential locally resolved velocities normalized to [-π,π][7]. The volumetric flow is calculated as the product between mean velocity and vessel cross-section area measured in the imaging plane rescaled by the cosine of the Doppler angle.

Supplementary Note 3

Doppler angle measurement. The mathematical expression for the Doppler angle has been developed from the calculation of the light path length difference at two scanning locations accounting for light refraction at the air-tissue interface (Supplementary Fig. 3c). It can be calculated from the geometrical length of the triangle's depth side in tissue Δz_t:

$$\tan(90 - \alpha) = \frac{\Delta z_t}{\Delta'}, \tag{9}$$

with Δ' the distance between both beams in tissue. The latter is obtained from the incidence angle in air θ and in tissue θ_t and Δ the beam distance in air given by the transverse extent of the triangle in air:

$$\Delta' = \Delta\frac{cos(\theta_t)}{cos(\theta)}. \tag{10}$$

The incidence angle in air can be calculated from the dimensions of the triangle in air:

$$cos(\theta) = \frac{\Delta}{\sqrt{\Delta^2+\Delta z_a{}^2}}, \tag{11}$$

with Δz_a the physical depth of the triangle in air. An expression for θ_t is obtained using Snell's law on the air-tissue interface ($n_t sin(\theta_t) = \sin(\theta)$), the dimensions of the triangle in air and the tissue refractive index n_t:

$$cos(\theta_t) = \sqrt{1-\sin(\theta_t)^2} = \sqrt{1-\frac{\sin(\theta)^2}{n_t{}^2}} = \sqrt{1-\frac{\Delta z_a{}^2}{n_t{}^2(\Delta^2+\Delta z_a{}^2)}} = \sqrt{\frac{n_t{}^2(\Delta^2+\Delta z_a{}^2)-\Delta z_a{}^2}{n_t{}^2(\Delta^2+\Delta z_a{}^2)}}. \tag{12}$$

The Doppler angle value can therefore be calculated using:

$$\tan(90-\alpha) = \frac{\Delta z_t}{\Delta'} = \frac{\Delta z_t}{\Delta}\frac{cos(\theta)}{cos(\theta_t)} = \frac{\Delta z_t}{\Delta}\frac{\Delta}{\sqrt{\Delta^2+\Delta z_a{}^2}} n_t\sqrt{\frac{(\Delta^2+\Delta z_a{}^2)}{n_t{}^2(\Delta^2+\Delta z_a{}^2)-\Delta z_a{}^2}} = \frac{\Delta z_t n_t}{\sqrt{n_t{}^2(\Delta^2+\Delta z_a{}^2)-\Delta z_a{}^2}}, \tag{13}$$

which also writes:

$$\tan(90-\alpha) = \frac{\Delta z_t n_t}{\sqrt{\Delta z_a{}^2(n_t{}^2-1)+(\Delta\cdot n_t)^2}}. \tag{14}$$

Supplementary References

1. Vakoc, B., Yun, S., de Boer, J., Tearney, G. & Bouma, B. Phase-resolved optical frequency domain imaging. *Opt. Express* **13**, 5483-5493 (2005).
2. Szkulmowski, M., Szkulmowska, A., Bajraszewski, T., Kowalczyk, A. & Wojtkowski, M. Flow velocity estimation using joint Spectral and Time domain Optical Coherence Tomography. *Opt. Express* **16**, 6008-6025 (2008).
3. Cheng, S., Hong Y. S., Zhang, G., Huang, C. H. & Huang, X. J. Measurement of the refractive index of biotissue at four laser wavelengths. I*nternational Society for Optics and Photonics - Photonics Asia*, 172-176 (2002).
4. Walther, J. & Koch, E. Relation of joint spectral and time domain optical coherence tomography (jSTdOCT) and phase-resolved Doppler OCT. *Opt. Express* **22**, 23129-23146 (2014).
5. Thévenaz, P., Ruttimann, U. E. & Unser, M. A Pyramid Approach to Subpixel Registration Based on Intensity. *IEEE Trans. Image Process.* **7**, 27-41 (1998).
6. Kass, M., Witkin, A. & Terzopoulos, D. Snakes: Active contour models. *Int. J. Comput. Vision* **1**, 321-331 (1988).
7. Szkulmowska, A., Szkulmowski, M., Kowalczyk, A. & Wojtkowski, M. Phase-resolved Doppler optical coherence tomography-limitations and improvements. *Opt. Lett.* **33**, 1425–1427 (2008).
8. Kasai, C., Namekawa, K., Koyano, A. & Omoto, R., Real-time two-dimensional blood flow imaging using an autocorrelation technique. *IEEE Trans. Son. Ultrason.* **32**, 458–464 (1985).
9. Chan, A. C., Lam, E. Y. & Srinivasan, V. J. Comparison of Kasai autocorrelation and maximum likelihood estimators for Doppler optical coherence tomography. *IEEE Trans. Med. Imaging* **32**, 1033–1042 (2013).
